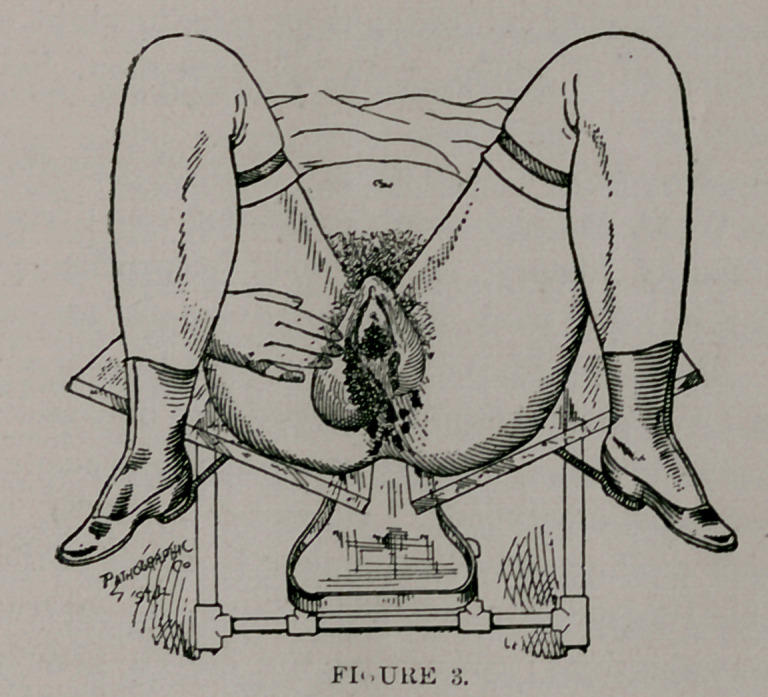# Phagedenic Chancroids

**Published:** 1894-09

**Authors:** 


PHAGEDENIC CHANCROIDS.—A. H., aged twenty-five a seamstress, was admitted to the Hospital April 3, 1894. She had, she said, been infected some three weeks before and had used vaseline. Examination revealed extension-phagedenic chancroids as shown in Figs. 2 and 3. The parts were thoroughly cleansed and dried; pure carbolic acid was then applied and the sores dusted with iodoform. At the next clinic, two days later, the patient was much improved, the swelling of the labia had subsided to some extent and

FIGURE 2.

most of the sores looked healthy. Those which did not were again smeared with pure carbolic acid and dusted with iodoform. Under

FIGURE 3.

this treatment the patient steadily improved and was finally discharged, fully recovered.—*Courier of Medicine.*

## Figures and Tables

**Figure 2. f1:**
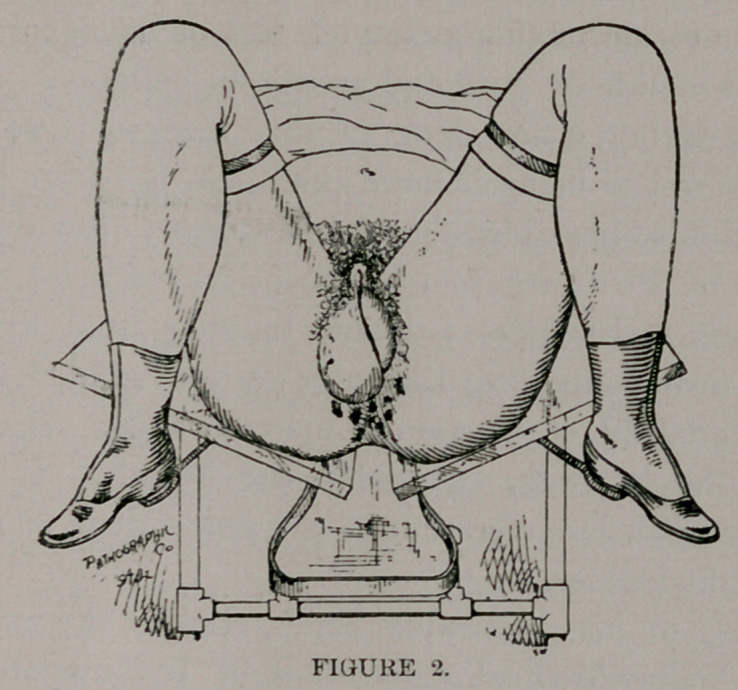


**Figure 3. f2:**